# Marker Assisted Breeding to Develop Multiple Stress Tolerant Varieties for Flood and Drought Prone Areas

**DOI:** 10.1186/s12284-019-0269-y

**Published:** 2019-02-18

**Authors:** Nitika Sandhu, Shalabh Dixit, B. P. M. Swamy, Anitha Raman, Santosh Kumar, S. P. Singh, R. B. Yadaw, O. N. Singh, J. N. Reddy, A. Anandan, Shailesh Yadav, Challa Venkataeshwarllu, Amelia Henry, Satish Verulkar, N. P. Mandal, T. Ram, Jyothi Badri, Prashant Vikram, Arvind Kumar

**Affiliations:** 1 International Rice Research Institute, DAPO Box 7777, Metro Manila, Philippines; 2 Punjab Agricultural University, Ludhiana, India; 3 ICAR Research Complex for Eastern Region, Patna, Bihar India; 4 Bihar Agricultural University, Sabour, Bihar India; 5 National Rice Research Program Hardinath, Dhanusha, Nepal; 6 ICAR-National Rice Research Institute, Cuttack, Odisha India; 7 International Rice Research Institute, South Asia Hub, ICRISAT, Patancheru, Hyderabad, India; 8 Indira Gandhi Krishi Vishwavidyalaya, Raipur, Chhattisgarh India; 9 Central Rainfed Upland Rice Research station, National Rice Research Institute, Hazaribagh, Jharkhand India; 10 ICAR-Indian Institute of Rice Research, Hyderabad, India; 11 International Maize and Wheat Improvement Centre (CIMMYT), Texcoco, Mexico

**Keywords:** Drought, Marker-assisted breeding, QTL pyramiding, Rice, Submergence, Varieties

## Abstract

**Background:**

Climate extremes such as drought and flood have become major constraints to the sustainable rice crop productivity in rainfed environments. Availability of suitable climate-resilient varieties could help farmers to reduce the grain yield losses resulting from the climatic extremities. The present study was undertaken with an aim to develop high-yielding drought and submergence tolerant rice varieties using marker assisted introgression of *qDTY*_*1.1*_, *qDTY*_*2.1*_, *qDTY*_*3.1*_ and *Sub1.* Performance of near isogenic lines (NILs) developed in the background of Swarna was evaluated across 60 multi-locations trials (MLTs). The selected promising lines from MLTs were nominated and evaluated in national trials across 18 locations in India and 6 locations in Nepal.

**Results:**

Grain yield advantage of the NILs with *qDTY*_*1.1*_ *+ qDTY*_*2.1*_ + *qDTY*_*3.1*_ + *Sub1* and *qDTY*_*2.1*_ + *qDTY*_*3.1*_ + *Sub1* ranged from 76 to 2479 kg ha^− 1^ and 396 to 2376 kg ha^− 1^ under non-stress (NS) respectively and 292 to 1118 kg ha^− 1^ and 284 to 2086 kg ha^− 1^ under reproductive drought stress (RS), respectively. The NIL, IR96322–34-223-B-1-1-1-1 having *qDTY*_*1.1*_ *+ qDTY*_*2.1*_ + *qDTY*_*3.1*_ + *Sub1* has been released as variety CR dhan 801 in India. IR 96321–1447-651-B-1-1-2 having *qDTY*_*1.1*_ + *qDTY*_*3.1*_ + *Sub 1* and IR 94391–131–358-19-B-1-1-1 having *qDTY*_*3.1*_ + *Sub1* have been released as varieties Bahuguni dhan-1′ and ‘Bahuguni dhan-2’ respectively in Nepal. Background recovery of 94%, 93% and 98% was observed for IR 96322–34-223-B-1-1-1-1, IR 96321–1447-651-B-1-1-2 and IR 94391–131–358-19-B-1-1-1 respectively on 6 K SNP Infinium chip.

**Conclusion:**

The drought and submergence tolerant rice varieties with pyramided multiple QTLs can ensure 0.2 to 1.7 t ha^− 1^ under reproductive stage drought stress and 0.1 to 1.0 t ha^− 1^ under submergence conditions with no yield penalty under non-stress to farmers irrespective of occurrence of drought and/or flood in the same or different seasons.

**Electronic supplementary material:**

The online version of this article (10.1186/s12284-019-0269-y) contains supplementary material, which is available to authorized users.

## Background

Increasing incidences of abiotic stresses under changing climate are major constraints to meet the ever-growing demand for food for rapidly escalating population and attain the global food security (Lesk et al. 2016). Drought and flood are the two most prevalent abiotic stresses reducing rice yield in the rainfed environments. Worldwide, drought and flood stresses have been reported to affect approximately 40 million hectares of total rice area at different crop stages producing negative impacts on plant growth, development and yield (Barnabás et al. 2008; Neeraja et al. 2007). Rainfed rice ecosystems in South Asia and Southeast Asia are the key hotspots for the occurrence of combination of drought and flood stresses (Dilley et al. 2005). High rainfall over short period during crop growth or low rainfall or early withdrawal of monsoon rains may bring flood or prolonged dry spell causing substantial reduction to crop yields (Lobell et al. 2011). Many times, both flood and drought may occur in the same season at different crop growth stages. In the coming years rainfed shallow lowland areas will face heavy precipitation during early crop growth stage leading to flood, and then dry period leading to drought at terminal stages. Variability in the pattern, intensity and frequency of rainfall with the changing climate are several of the factors leading to unpredictable occurrence of drought and flood conditions. These adverse conditions are causing crop failures, volatility in economic growth and making it harder for the small and marginal farmers to move up from the persistent poverty (Mottaleb et al. 2015).

In Nepal, about 50% and in India more than 33% of the total cropland is dedicated to cultivation of rice (Pandey and Bhandari 2007; Gumma et al. 2011). Large area in the rainfed rice-growing ecologies India and Nepal are vulnerable to submergence and drought (Dar et al. 2014). More than 7.3 and 0.27 million hectares of rainfed lowland rice ecologies in India and Nepal respectively, are affected by drought stress (Pandey and Bhandari 2008). Over 5 million hectares of rice land in India is prone to submergence, leading to a paddy loss of 4 million tons per year which is otherwise enough to provide food to 30 million people (Mottaleb et al. 2015).

The rice varieties such as Swarna, Samba Mahsuri, IR64 and MTU1010 are popular among the farmers in India because of their high yield, preferred grain quality traits and higher market value. However, most of these rice varieties are extremely sensitive to drought and submergence, leading to high yield losses every year in regions of their cultivation. The traditional varieties cultivated before the development of semi-dwarf green revolution varieties are less sensitive to drought and flood but poor in yield and grain quality. Introgression of drought and flood tolerance into existing popular rice varieties has been an effective approach to cope with the effects of drought and submergence and reduce yield losses under drought and flood.

In past few decades, efforts have been devoted at the International Rice Research Institute (IRRI) in identifying major genes/QTLs (Kumar et al. 2014), developing selection strategies (Kumar et al. 2018) and understanding the genetics of grain yield under drought in rice (Sandhu and Kumar 2017). Major effect drought QTLs explaining large proportion of the phenotypic variance for grain yield such as *qDTY*_*1.1*_ (Vikram et al. 2011; Ghimire et al. 2012; Sandhu et al. 2014), *qDTY*_*2.1*_ (Venuprasad et al. 2009; Sandhu et al. 2014) and *qDTY*_*3.1*_ (Dixit et al. 2014; Venuprasad et al. 2009) have been identified. These QTLs in synergistic combinations of two to three together can provide grain yield advantage of 0.8 to 1.0 t ha^− 1^ under reproductive stage drought stress (Sandhu and Kumar 2017).

A major effect QTL *Sub1* from a landrace FR13A explaining phenotypic variation of 69% (Xu and Mackill 1996; Septiningsih et al. 2015) providing tolerance to two weeks of complete submergence has also been identified. The submergence tolerance of many mega varieties such as Swarna (Neeraja et al. 2007), Ciherang (Septiningsih et al. 2015; Toledo et al. 2015) and PSB Rc18 (Septiningsih et al. 2015) were improved using marker-assisted backcross breeding of *Sub1*.

The rice varieties combining drought and submergence can provide yield insurance to farmers in regions exposed to occurrence of drought or submergence or both in the rainfed ecosystems. In this study, the strategy of marker assisted backcross selection in the early stages combined with phenotypic selection at later stage of development has been followed. The present study was conducted with the aim to develop the drought-submergence tolerant near-isogenic lines (NILs) in the background of Swarna combining high yield under non-stress, drought and submergence conditions with preferred grain quality traits. The objectives of the present study include (1) to introgress *qDTY*_*1.1*_, *qDTY*_*2.1*_ and *qDTY*_*3.1*_ with *Sub1* in the background of Swarna (2) to study the interactions between the drought QTLs and *Sub1* in terms of performance under drought or submergence when introgressed together (3) to study the performance of breeding lines with different combinations of DTY QTLs and *Sub1* on grain yield under reproductive stage drought stress (RS), submergence stress (Sub) and non-stress conditions (NS).

## Results

### Single trial analysis

The developed NILs were screened at IRRI and in national trials in India (Hyderabad, Sabour, Faizabad, Madhepura, Dhangain, Patna, Varanasi, Tripura, Cuttack and Raipur) and Nepal (Hardinath and Nepalgunj). A total of 60 multi-locations experiments were conducted (Additional file 1: Table S1). The mean grain yield ranged from 2037 to 9848 kg ha^− 1^ under NS (non-stress), 175 to 6739 kg ha^− 1^ under RS (reproductive stage drought stress) and 1278 to 8068 kg ha^− 1^ under submergence conditions (Additional file 1: Table S1). The days to 50% flowering (DTF) ranged from 83 to 116 days under NS, 85 to 118 days under RS and 83 to 128 under submergence conditions. The plant height varied from 84 to 110 cm under NS, 54 to 99 cm under RS and 74 to 115 cm under submergence conditions (Additional file 1: Table S1).

### Performance of Swarna lines introgressed with drought and submergence QTLs

**Fig. 1:**
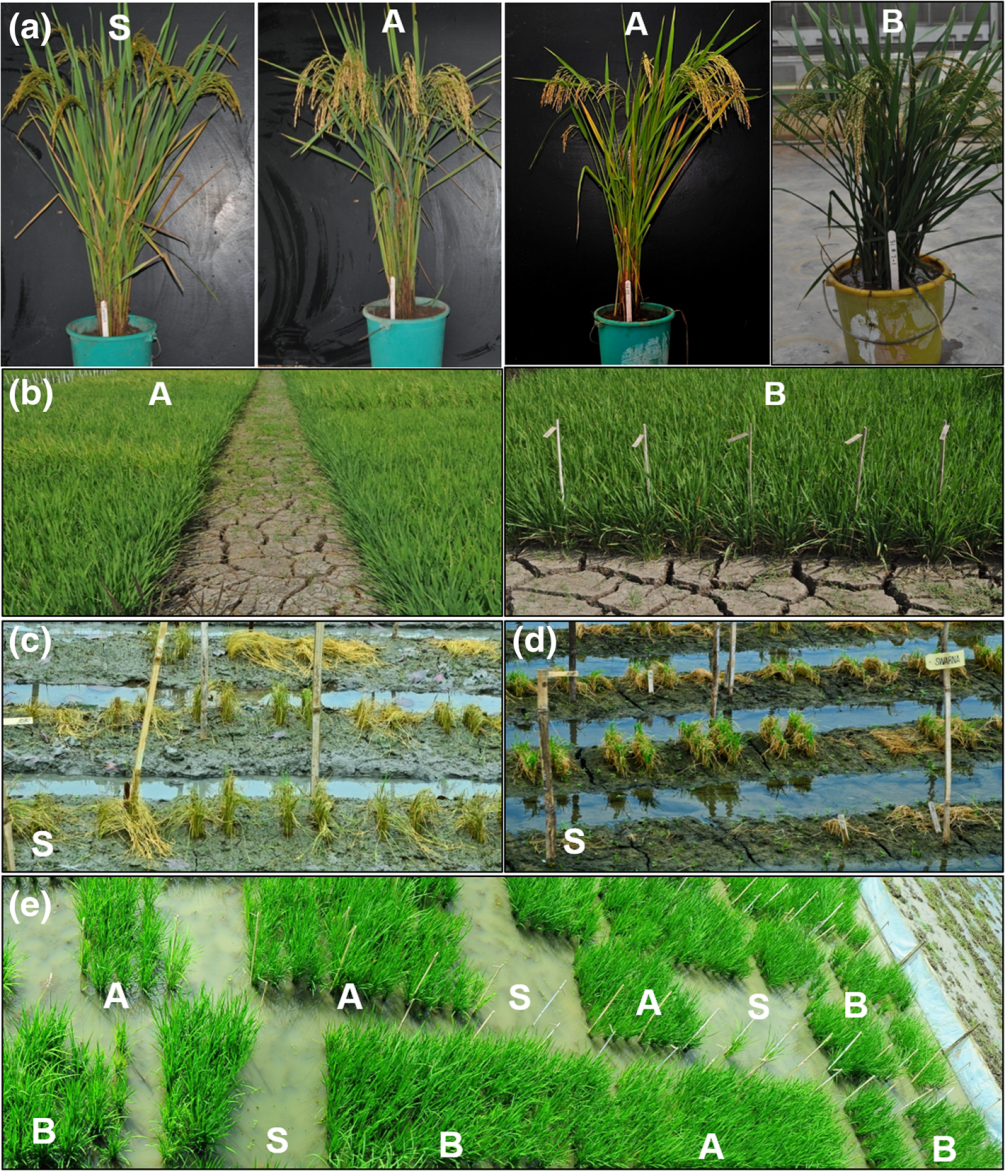
**a** The morphological differences between Swarna and introgressed NILs; **b** field view of introgressed NILs under reproductive stage drought stress condition; submergence tolerance of introgressed NILs compared to Swarna (**c**) one day after draining; **d** six day after draining; **e** recovery of introgressed NILs compared to Swarna one month after draining. S: Swarna, A: introgressed NIL with *qDTY*_*2.1*_ + *qDTY*_*3.1*_ + *Sub1* B: introgressed NIL with *qDTY*_*1.1*_ + *qDTY*_*2.1*_ + *qDTY*_*3.1*_ + *Sub1*

The NILs in Swarna background with either single or multiple QTL produced grain yield advantage over Swarna (Additional file 1: Table S2; Fig. 1). The NILs with *qDTY*_*1.1*_ *+ qDTY*_*2.1*_ + *qDTY*_*3.1*_ + *Sub1* and *qDTY*_*2.1*_ + *qDTY*_*3.1*_ + *Sub1* showed grain yield advantage that ranged from 76 to 2479 kg ha^− 1^ and 396 to 2376 kg ha^− 1^ over Swarna under NS respectively. Grain yield advantage of 292 to 1118 kg ha^− 1^ and 284 to 2086 kg ha^− 1^ was observed in *qDTY*_*1.1*_ *+ qDTY*_*2.1*_ + *qDTY*_*3.1*_ + *Sub1* and *qDTY*_*2.1*_ + *qDTY*_*3.1*_ + *Sub1* NILs respectively over Swarna under RS (Additional file 1: Table S2). The grain yield advantage of NILs having *qDTY*_*1.1*_ + *qDTY*_*3.1*_ + *Sub1* QTL combination ranged from 37 to 1350 kg ha^− 1^ and 95 to 629 kg ha^− 1^ over Swarna under NS and RS, respectively. In addition, the two QTL classes (*qDTY*_*1.1*_ *+ qDTY*_*2.1*_ + *qDTY*_*3.1*_ + *Sub1* and *qDTY*_*2.1*_ + *qDTY*_*3.1*_ + *Sub1*) showed consistently higher performance than other QTL classes under both NS and RS across advancing generations (Additional file 1: Table S2). The NILs with days to 50% flowering less than that of Swarna and Swarna-Sub1 under NS and RS were identified (data not shown). Most of the NILs showed similar plant height as that of Swarna and Swarna-Sub1 under NS and less under RS (data not shown).

### QTL × environment

The variance component for QTL × environment interaction was significant at all stress levels; non-stress, moderate drought stress and severe drought stress. A significant environment variance component was observed in the case of non-stress experiments. The QTL main effect was significant only under severe drought stress (Table 1).

**Table 1:** The mixed model (REML) parameters of the combined analysis for all three stresses levels

Stress	Cov Parm	Estimate	Pr > Z
Non-stress	QTL	69,808	0.0594
	environment	2,802,695	0.0007
	QTL × environment	231,386	0.0009
	Residual	585,608	<.0001
Moderate drought stress	QTL	5971.56	0.4548
	environment	136,417	0.1539
	QTL × environment	1418.58	0.4891
	Residual	428,228	<.0001
Severe drought stress	QTL	107,741	0.0013
	environment	90,708	0.1047
	QTL × environment	68,940	0.0099
	Residual	81,811	<.0001
Tests of (Fixed Effects)			
Stress	Effect	F Value	Pr > F
Non-stress	QTL	1.25	0.2468
Moderate drought stress	QTL	1.16	0.4285
Severe drought stress	QTL	1.64	0.0781

The selected NILs showed significant yield advantage over recipient parent Swarna under reproductive stage drought indicating capture of positive interaction between QTL × QTL or QTL × genetic background utilizing strategy combining genotypic and phenotypic selection for grain yield under NS and RS.

### Performance of Swarna NILs with different QTL combinations across different locations

The tested NILs across all location and trials (Additional file 1: Table S1) were pooled together and categorised based on QTL combinations into three different stress levels; non-stress, moderate drought stress and severe drought stress. The NILs with *qDTY*_*1.1*_ *+ qDTY*_*2.1*_ + *qDTY*_*3.1*_ + *Sub1, qDTY*_*1.1*_ *+ qDTY*_*3.1*_ + *Sub1* and *qDTY*_*3.1*_ + *Sub1* had shown consistent grain yield advantage across different locations and seasons (Table 2).

**Table 2:** The mean grain yield performance of Swarna NILs with different QTL combinations across different locations at three different stress levels

QTL	Non-stress				Moderate drought stress				Severe drought stress			
	No of entries	No. of observations	Mean	Std err	No of entries	No. of observations	Mean	Std err	No of entries	No. of observations	Mean	Std err
*qDTY*_*1.1*_ + *Sub1*	5	24	6336	448.9	4	10	2355	248.0	5	9	1378	279.9
*qDTY*_*2.1*_ + *Sub1*	1	4	7217	640.6					1	2	1585	457.1
*qDTY*_*3.1*_	3	11	7285	488.6	2	3	2903	368.3	3	6	1662	298.0
*qDTY*_*3.1*_ + *Sub1*	6	39	6421	377.7	6	17	2502	334.7	6	14	1337	262.9
*qDTY*_*1.1*_ + *qDTY*_*2.1*_ + *Sub1*	6	25	6633	432.0	3	7	2488	262.5	4	8	1426	312.2
*qDTY*_*1.1*_ + *qDTY*_*3.1*_ + *Sub 1*	13	74	6403	396.3	10	24	2552	264.5	12	31	1201	216.3
*qDTY*_*2.1*_ + *qDTY*_*3.1*_ + *Sub1*	4	26	6752	445.5	3	10	2558	282.6	4	10	1161	300.1
*qDTY*_*1.1*_ + *qDTY*_*2.1*_ + *qDTY*_*3.1*_	6	15	6798	461.7	3	3	2935	440.1	3	6	1134	331.5
*qDTY*_*1.1*_ + *qDTY*_*2.1*_ + *qDTY*_*3.1*_ + *Sub1*	20	86	6711	388.1	9	18	2902	256.2	11	20	1599	239.8
Swarna-Sub1	1	68	6112	422.3	1	8	1678	332.1	1	21	882	255.2

Total number of times entries tested in different trials across locations and seasons, *Std err* standard error

### Selection of promising NILs

Seeds of the selected promising lines (48 lines in 2014WS) were multiplied and shared with NARES (National Agricultural Research and Extension Systems) partners for further screening across multi locations under NS, RS and submergence conditions. The selected NILs showed grain yield advantage over both Swarna and Swarna-Sub1 under NS, RS and submergence stress conditions. Under NS and RS, IR 94391-131-358-19-B-1-1-1 showed highest grain yield advantage followed by IR 96322-34-223-B-1-1-1 and IR 96322-34-127-B-1-1-1 over Swarna (Table 3). Similar pattern of grain yield advantage was observed for NILs over Swarna-Sub1 under NS and RS (Table 3). The three selected promising NILs had shown grain yield advantage ranged from 109 to 2382 kg ha^− 1^ over Swarna and 99 to 2448 kg ha^− 1^ over Swarna-Sub1 under NS (Table 3). Under RS, the grain yield advantage of three selected promising NILs ranged from 415 to 1933 kg ha^− 1^ over Swarna and 38 to 1786 kg ha^− 1^ over Swarna-Sub1 (Table 4). The selected NILs had shown consistent performance across different locations and seasons. Under submergence conditions, the NIL IR 94391-131-358-19-B-1-1-1 had shown highest grain yield advantage followed by IR 96322-34-127-B-1-1-1 and IR 96322-34-223-B-1-1-1 over Swarna and Swarna-Sub1 both.

**Table 3:** The mean grain yield (kg ha^−1^) performance of selected promising lines across different locations and seasons under non-stress conditions

Season	QTLs (% Background recovery)	2014 WS	2015 DS	2015 DS	2015 WS	2015 WS	2015 WS	2015 WS	2015 WS	2015 WS	2015 WS	2016 WS	2016 WS	2016 WS	Combined mean
Location		IRRI SAH	IRRI-HQ	IRRI SAH	IRRI SAH	Sabour	Patna	Varanasi	Hazaribagh	Hardinath	IRRI-HQ	Patna	Hazaribagh	IRRI-HQ	
Targeted stress		NS	NS	NS	NS	NS	NS	NS	NS	NS	NS	NS	NS	NS	
Achieved stress		NS	NS	NS	NS	NS	NS	NS	NS	NS	NS	NS	NS	NS	
IR 96321-1447-651-B-1-1-2	*qDTY*_*1.1*_ + *qDTY*_*3.1*_ + *Sub 1* (93%)	6675	6474	6737	5772	8185	6900	9608	6405	4973	5695	7896	5789	3275	6491
IR 96321-558-563-B-2-1-1	*qDTY*_*3.1*_ + *Sub 1* (89%)	6886	5211	5279	5747	6845	8407	9750	5514	4804	5013	7175	6368	3888	6222
IR 96322-34-260-B-5-1-1	*qDTY*_*1.1*_ + *qDTY*_*2.1*_ + *qDTY*_*3.1*_ + *Sub 1* (90%)	6101	6235	6977	6039	9167	8420	10,476	6557	4682	5387	6757	5962	4046	6677
IR 96322-34-223-B-1-1-1	*qDTY*_*1.1*_ + *qDTY*_*2.1*_ + *qDTY*_*3.1*_ + *Sub 1* (94%)	6340	6218	7632	6114	8378	7666	10,261	6064	5201	5595	–	6787	3687	6662
IR 96321-558-257-B-5-1-2	*qDTY*_*3.1*_ + *Sub 1* (92%)	6068	5706	6741	5557	7887	7753	8476	4649	4278	4696		5589	–	6127
IR 96321-558-563-B-2-1-3	*qDTY*_*3.1*_ + *Sub 1* (93%)	6627	5719	6949	6196	9494	7113	10,333	5814	4797	5155	–	4537	–	6612
IR 94391-131–358-19-B-1-1-1	*qDTY*_*3.1*_ + *Sub 1* (98%)	6294	7764	6867	6107	–	–	–	–	5572	–	–	–	–	6521
IR 94391-131–358-19-B-6-1-4	*qDTY*_*3.1*_ + *Sub 1* (92%)	6207	7974	8478	–	–	–	–	–	–	–	–	–	–	7553
IR 96322-34–127-B-1-1-1	*qDTY*_*1.1*_ + *qDTY*_*3.1*_ + *Sub 1* (95%)	6611	5925	6660	–	–	–	–	–	–	–	–	–	–	6399
Swarna-Sub1	*Sub1*	6049	5316	6377	6008	8277	7307	9738	4015	4622	5136	7561	6170	3301	6144
Swarna	–	6021	5382	5904	5788	8063	7547	10,128	4905	3890	5486	7038	5914	2927	6076
Trial mean		6186	6787	6017	5968	8375	7743	9848	5651	4523	4984	8921	5940	4010	6535
LSD_0.05_		1406	1128	1401	825	677	632	397	553	811	780	604	625	680	809.15
Trial H		0	0.86	0.89	0.37	0.21	0.09	0.94	0.89	0.54	0.80	0.90	0.81	0.79	0.62

*DS* dry season*, WS* wet season*, NS* non-stress*,* IRRI HQ IRRI headquarter (Philippines)*,* IRRI SAH IRRI South Asia Hub (Hyderabad)*, H* heritability

**Table 4:** The mean grain yield (kg ha^−1^) performance of selected promising lines across different locations and seasons under moderate and severe reproductive stage drought stress (RS) and submergence conditions (Sub)

Season	QTLs	2014 WS	2015 WS	2015 WS	2014 WS	2015 DS	2016 WS	2016 WS	2014 WS	2014 WS	Combined mean		
Location		Hardinath	Patna	Hardinath	Hardinath	IRRI-HQ	Raipur	Nepal gunj	Hardi nath	Nepal gunj			
Targeted stress		NS	RS	RS	RS	RS	RS	RS	Sub	Sub	RS		Sub
Achieved stress		Moderate drought stress	Moderate drought stress	Moderate drought stress	Severe drought stress	Severe drought stress	Severe drought stress	Severe drought stress	Sub	Sub	Moderate drought stress	Severe drought stress	
IR 96321-1447-651-B-1-1-2	*qDTY*_*1.1*_ + *qDTY*_*3.1*_ + *Sub 1* (93%)	2799	3796	3300	1155	1525	836	909	1351	763	3298	1106	1057
IR 96321-558-563-B-2-1-1	*qDTY*_*3.1*_ + *Sub 1* (89%)	2306	3542	2033	1298	1635	1681	763	1664	1379	2627	1344	1522
IR 96322-34-260-B-5-1-1	*qDTY*_*1.1*_ + *qDTY*_*2.1*_ + *qDTY*_*3.1*_ + *Sub 1* (90%)	2378	2359	1100	1110	1758	1236	1539	1151	715	1946	1411	933
IR 96322-34-223-B-1-1-1	*qDTY*_*1.1*_ + *qDTY*_*2.1*_ + *qDTY*_*3.1*_ + *Sub 1* (94%)	3068	3589	2600	1105	1566	1361	–	1383	1359	3086	1344	1371
IR 96321-558-257-B-5-1-2	*qDTY*_*3.1*_ + *Sub 1* (92%)	2548	2017	2333	845	1679	–	–	1105	1532	2299	1262	1319
IR 96321-558-563-B-2-1-3	*qDTY*_*3.1*_ + *Sub 1* (93%)	2393	3383	2800	1163	2077	–	–	1662	1087	2859	1620	1375
IR 94391-131–358-19-B-1-1-1	*qDTY*_*3.1*_ + *Sub 1* (98%)	3390	–	2900	1405	2307	–	–	1989	1311	3145	1856	1650
IR 94391-131–358-19-B-6-1-4	*qDTY*_*3.1*_ + *Sub 1* (92%)	3133	–	–	1133	1870	–	–	1500	–	3133	1502	1500
IR 96322-34-127-B-1-1-1	*qDTY*_*1.1*_ + *qDTY*_*3.1*_ + *Sub 1* (95%)	2221	–	–	1103	1349	–	–	1650	–	2221	1226	1650
Swarna-Sub1	*Sub1*	2183	2658	1700	1008	521	764	789	1081	1154	2180	771	1118
Swarna	–	1457	2739	1867	688	755	684	575	173	150	2021	676	162
Trial mean		2447	2987	2353	1072	1510	1267	792	1290	1231	2596	1160	1261
LSD_0.05_		1437	709	908	340	585	425	630	320	554	887	554	437
Trial H		0.53	0.92	0.63	0.52	0.81	0.95	0.64	0.36	0.40	0.69	0.73	0.38

*DS* dry season*, WS* wet season*, NS* non-stress*, RS* reproductive stage drought stress*, Sub* Submergence stress DS dry season*,* IRRI HQ IRRI headquarter (Philippines)*, H* heritability

In addition, other NILs such as IR 96321-1447-651-B-1-1-2, IR 96321-558-563-B-2-1-3 and IR 94391-131-358-19-B-6-1-4 had shown grain yield advantage that ranged from 72 to 1600 kg ha^− 1^, 155 to 1556 kg ha^− 1^ and 38 to 828 kg ha^− 1^ over Swarna-Sub1, respectively under RS (Table 4). The promising NILs had shown better survival percentage over Swarna-Sub1 under 13 and 21 days of submergence. The survival percentage ranged from 83 to 96% after 14 days of submergence period and 75 to 92% after 21 days of submergence (Table 5).

**Table 5:** Survival percentage (%) of NILs screened at Patna in 2015DS under submergence conditions

Designation	Background recovery (%)	Survival %	
		14 days^a^	21 days^a^
IR 96321-315-323-B-3-1-3	93	96	82
IR 96321-315-323-B-3-1-1	93	90	82
IR 96321-558-209-B-6-1-1	98	83	82
IR 96321-558-257-B-4-1-2	91	92	92
IR 96321-1099-227-B-3-1-3	90	94	89
IR 96321-558-563-B-2-1-3	95	87	75
IR 96321-558-563-B-2-1-1	89	88	81
Swarna Sub 1	–	85	54
Swarna	–	58	6
LSD_0.05_	–	7	26

^a^Submergence period, date of seeding 17-2-2015, Date of first-time submergence 27-03-2015, date of de-submergence 09-04-2015, date of second time submergence 14-05-2015, Date of de-submergence 04-06-2015

### Performance of selected NILs under national trials

**Fig. 2:**
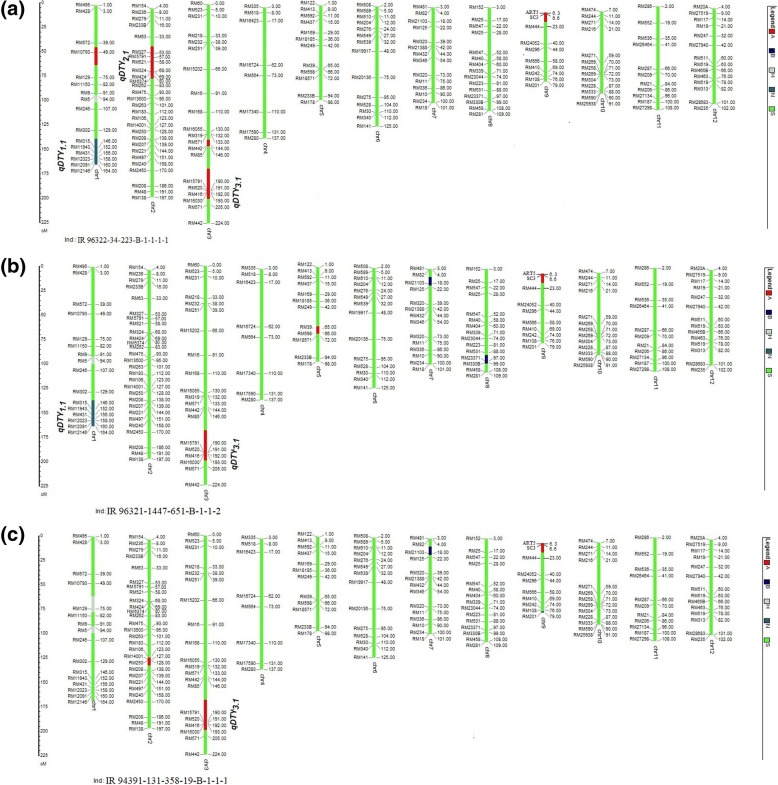
Graphical representation of the background recovery of NILs (**a**) IR 96322-34-223-B-1-1-1-1 (**b**) IR 96321-1447-651-B-1-1-2 (**c**) IR 94391-131-358-19-B-1-1-1 using molecular marker data was performed in Graphical Genotypes (GGT 2.0) software (Van Berloo 1999). Apo parent allele, N22 allele, Swarna-Sub1 allele, heterozygous allele and any other allele, were scored as ‘A’, ‘N’, ‘S’, ‘H’, and ‘B’, respectively

**Fig. 3:**
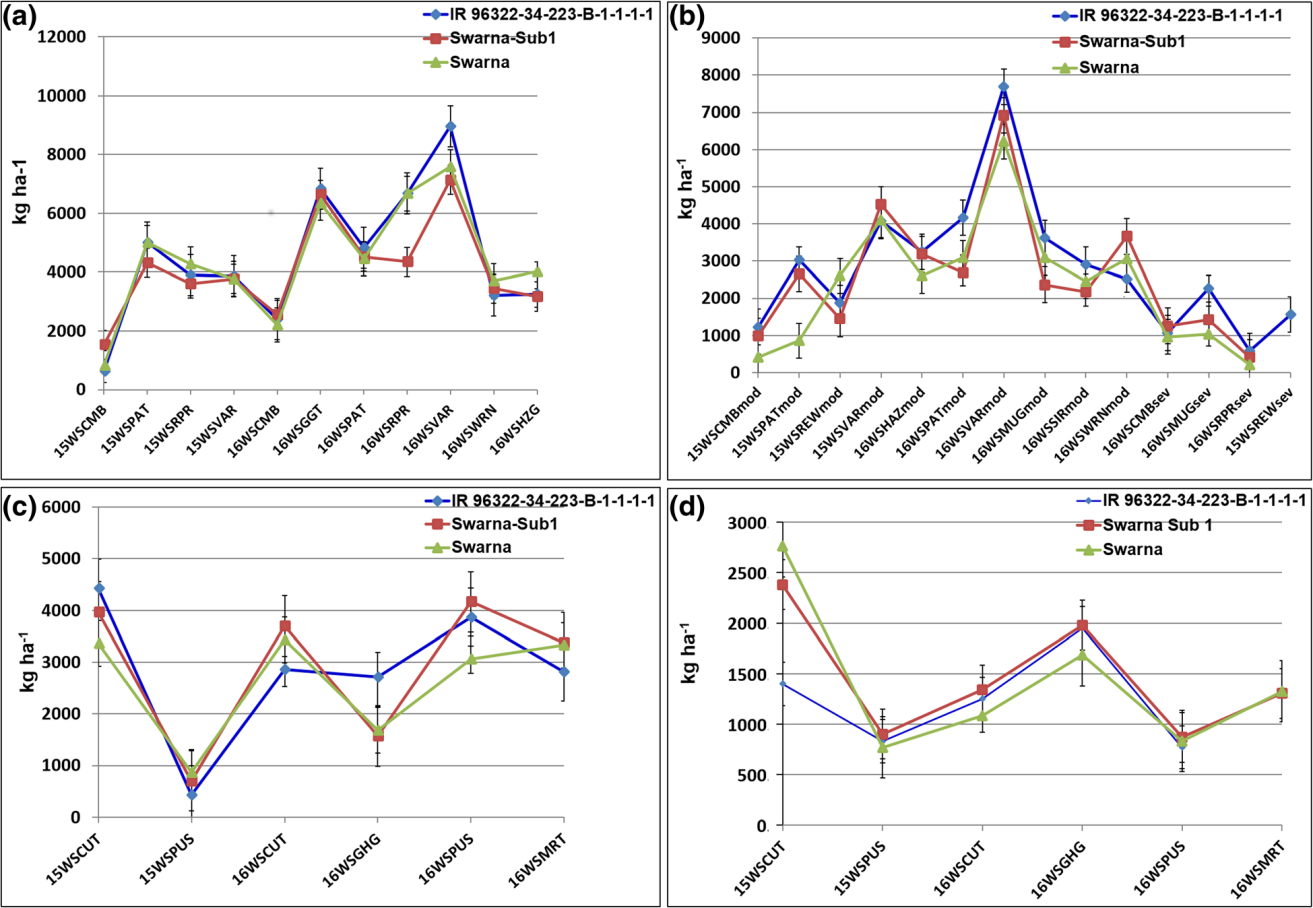
The grain yield performance of IR 96322-34-223-B-1-1-1-1 in comparison with Swarna-Sub1 and Swarna (**a**) under NS {control for RS}; **b** under RS; **c** under NS {control for Submergence}; **d** under Submergence in 2015WS and 2016WS in different locations under AICRIP trials in India. CMB: Coimbatore, CUT: Cuttack, GGT: Ghaghraghat, HZG: Hazaribag, MUG: Mugad, MRT: Maruteru, PAT: Patna, PUS: Pusa, RPR: Raipur, REW: Rewa, SIR: Sirsi, VAR: Varanasi, WRN: Warangal, DS: dry season, WS: wet season, NS – non-stress, RS: reproductive stage drought stress, mod: moderate drought stress, sev: severe drought stress

The selected nine promising lines were nominated under All India Co-ordinated Rice Improvement Program (AICRIP) in India and in Nepal for varietal release. The 156 polymorphic SSRs markers were used to study the percentage background recovery of selected nine promising lines. The percentage background recovery ranged from 89 to 98% (Tables 3, 4 and 5). In addition, out of the nine selected lines, 5 promising lines with good performance over locations (IR 96321-1447-651-B-1-1-2, IR 96322-34-223-B-1-1-1, IR 96321-558-257-B-5-1-2, IR 96321-558-563-B-2-1-3 and IR 96322-34-127-B-1-1-1) were genotyped using 6 K SNP chip. Among these five, IR 96322-34-223-B-1-1-1-1 in India and IR 94391-131-358-19-B-1-1-1 and IR 96321-1447-651-B-1-1-2 in Nepal have been released/identified for release as varieties. The graphical representation of the allelic distribution of the three NILs released as varieties (IR 96322-34-223-B-1-1-1-1, IR 96321-1447-651-B-1-1-2 and IR 94391-131-358-19-B-1-1-1) across 12 rice chromosomes is presented in Fig. 2. The performance of IR 96322–34-223-B-1-1-1-1 in comparison with Swarna-Sub1 and Swarna across 2015WS and 2016WS at 18 different locations under NS, RS and submergence conditions in AICRIP is presented in Fig. 3. The Swarna-Sub1 NIL, IR 96322–34-223-B-1-1-1-1 having *qDTY*_*1.1*_ *+ qDTY*_*2.1*_ + *qDTY*_*3.1*_ + *Sub1* has been released for cultivation in India in 2017 as variety under name ‘CR dhan 801’. It has maturity duration of 125 to 130 days, suitable to be grown under shallow to medium lowland areas, have short bold grain type, plant height of 80 to 98 cm, and an average yield of 5.0 to 5.5 t ha^− 1^.

**Fig. 4:**
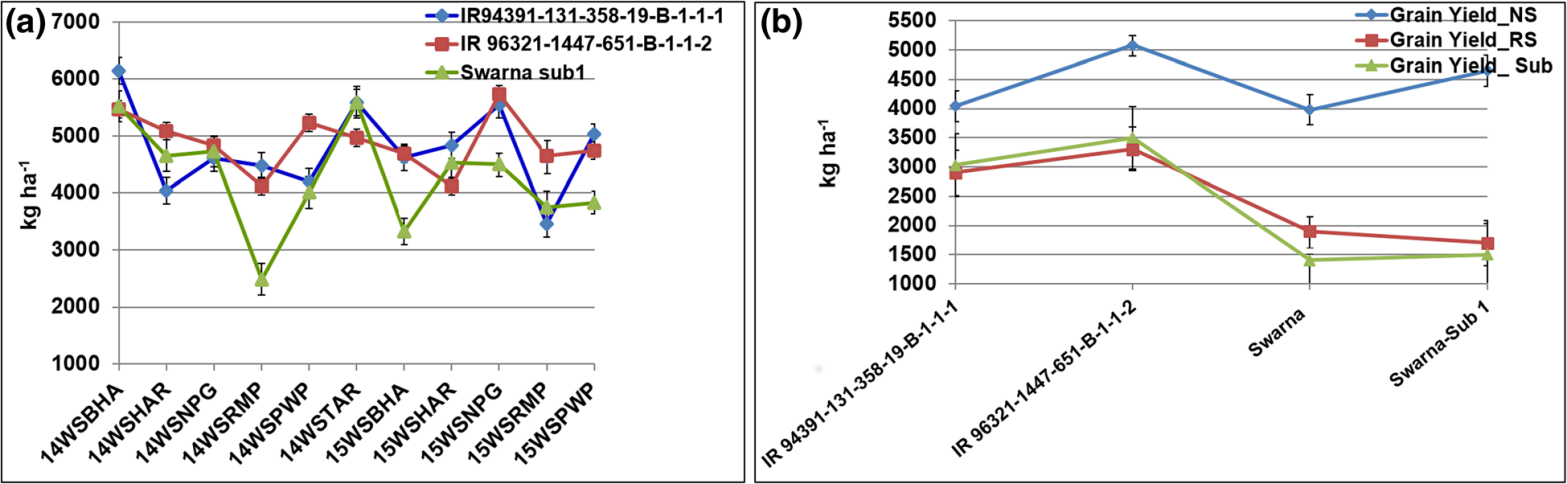
The mean grain yield performance of NILs in comparison with (**a**) Swarna-Sub1 across multilocational trials in Nepal under NS; **b** Swarna and Swarna-Sub1 in 2015WS at Hardinath under NS, RS and submergence conditions. BHA: Bhairahawa; HAR: Hardinath; NPG: Nepalgunj; RMP: Rampur; PWP: Parwanipur; TAR: Tarahara NS: non-stress, RS: reproductive stage drought stress, Sub: Submergence.

The performance of IR 94391-131-358-19-B-1-1-1 and IR 96321-1447-651-B-1-1-2 in comparison with Swarna-Sub1 in 2014WS and 2015WS at 6 different locations in Nepal under NS in national trials is shown in Fig. 4a. The performance of IR 94391-131-358-19-B-1-1-1 and IR 96321-1447-651-B-1-1-2 in comparison with Swarna and Swarna-Sub1 in 2015WS at Hardinath under NS, RS and Submergence conditions is presented in Fig. 4b. The Swarna-Sub1 NILs, IR 96321-1447-651-B-1-1-2 having *qDTY*_*1.1*_ + *qDTY*_*3.1*_ + *Sub 1* and IR 94391-131-358-19-B-1-1-1 having *qDTY*_*3.1*_ + *Sub 1* have been released as varieties in Nepal in 2017 under name ‘Bahuguni dhan-1’ and ‘Bahuguni dhan-2’, respectively. Both these varieties have maturity duration of 130 to 135 days, plant height of 95 to 105 cm, and shown average grain yield of 4.5 to 5.5 t ha^− 1^. The grain quality parameters of released varieties in comparison to Swarna and Swarna-Sub1 are presented in Table 6. Bahuguni Dhan 1 is fine grain and Bahuguni Dhan 2 has medium grain type as Swarna. The background recovery of 94%, 93% and 98% was observed for IR 96322-34-223-B-1-1-1-1, IR 96321-1447-651-B-1-1-2 and IR 94391-131-358-19-B-1-1-1, respectively on 6 K SNP Infinium chip.

**Table 6:** Grain quality parameters of the varieties released in India and Nepal in Swarna background in 2017

Designation	Variety name	Chalkiness	Grain length	Grain width	Amylose content	Weight milled rice	% milled rice	Weight head rice	% head rice	Crude Ash (%)	kjeldahl N (%)
IR 96322-34-223-B-1-1-1-1	CR dhan 801	7.4	5.48	2.32	27.4	85.3	68.2	70.7	56.5	–	–
IR 96321-1447-651-B-1-1-2	Bahuguni dhan-1	2.9	5.50	2.23	25.5	82.9	66.3	65.8	52.6	1.33	1.19
IR 94391-131-358-19-B-1-1-1	Bahuguni dhan-2	1.8	5.69	2.34	26.9	86.9	69.5	75.3	60.2	1.31	1.17
Swarna-Sub1	–	3.2	5.76	2.23	26.8	82.9	66.3	73.5	58.8	–	–
Swarna	–	3.9	5.58	2.29	27.9	82.1	65.6	71.1	56.8	–	–

## Discussion

Farmers in drought and submergence prone areas are mainly cultivating the abiotic stress susceptible rice varieties primarily due to their good grain yield potential and market-driven grain quality traits. The performance of these varieties is generally good during the non-drought-submergence affected years (Dar et al. 2018). However, during the natural calamities, large reduction in grain yield is observed in these varieties due to their inability to survive and yield under drought or submergence or both. Some of the rice growing regions are equally vulnerable to both drought and submergence. There is a need to look at the problem of farmers in a holistic way to improve the resilience of farmer’s livelihood so that food and other basic needs can be met on a sustainable basis. Development of dual flood and drought tolerant rice varieties lead to the overall increase in the farm output, farmer’s income and will improve the livelihood systems of rice farming communities.

The introduction of marker-assisted breeding in agriculture has provided new opportunities towards the introgression of identified trait associated genes/QTLs in several popular rice varieties (Dixit et al. 2017; Shamsudin et al. 2016). The grain yield advantage over Swarna and Swarna-Sub1 under RS drought stress and submergence, of those NILs possessing earlier identified major and consistent-effect QTLs, *qDTY*_*1.1*_ (Vikram et al. 2011; Ghimire et al. 2012; Sandhu et al. 2014), *qDTY*_*2.1*_ (Venuprasad et al. 2009; Sandhu et al. 2014), *qDTY*_*3.1*_ (Dixit et al. 2014; Venuprasad et al. 2009) and *Sub1* (Neeraja et al. 2007) in the current marker-assisted backcrossing breeding programs indicates the suitability of these loci in improving drought-submergence tolerance in the Swarna background. In addition, the selected NILs showed significant grain yield advantage under NS as the selection for grain yield under both NS and RS was made across generation advancement. IR 96322-34-223-B-1-1-1 showed grain yield advantage of 2 to 54% under NS (Table 3) and 1 to 17% under moderate to severe drought stress (Table 4) over the combined mean across seasons and locations. IR 94391-131-358-19-B-1-1-1 showed grain yield advantage of 5 to 19% under NS (Table 3) and 8 to 24% under moderate to severe drought stress (Table 4) over the combined mean across seasons and locations. Similarly, IR 94391-131-358-19-B-1-1-1 showed grain yield improvement of 3 to 48% under NS (Table 3) and 4 to 38% under moderate to severe drought stress (Table 4) over the combined means across seasons and locations. The improvement of 0.8 to 1.0 t ha^− 1^ in the yield of pyramided lines in different backgrounds as reported earlier (Vandana, IR64, MTU1010, TDK1, and MRQ74) (Kumar et al. 2014; Sandhu and Kumar 2017) as well as in the current study validates the success of QTL introgression in improving grain yield and tolerance to multiple stresses. The developed NILs were previously shown to perform well across different severity/intensity of drought and in regions with different conditions/ soil types (Singh et al. 2017). Some of the selected NILs showed early days to flowering than Swarna under NS (data not shown) and this may have resulted from the linkages of the *qDTYs* with earliness (Vikram et al. 2016). The plant height of most of the selected NILs were similar to the PHT of Swarna under NS but higher under RS (data not shown), this may be due to their increased ability to produce more biomass under RS. The linkage has been successfully broken and semi-dwarf, medium duration NILs in Swarna background have been developed.

QTL × environment interaction is a phenomenon in which QTL effects may significantly differ across environments. In the present study, the significant QTL × environment interaction for all the stress levels and the release of varieties with different combinations of QTLs in different ecosystems indicating the role of environment in influencing the effect of QTLs. The expression of introgressed genomic loci affecting grain yield and yield contributing components is influenced by the environment and the same loci may have differential effects in different ecosystems, signifying strong QTL × environment interaction (Xing et al. 2002). In the present study, the differential performance of NILs with same QTL combination in same or different ecosystem could be due to unknown QTL x genetic background interactions. There is urgent need to identify such epistatic gene interactions which complicates the genotype phenotype relationship of complex trait (Carlborg and Haley 2004) such as drought and submergence.

The phenotypic screening of NILs under different stresses; non-stress, drought stress and submergence conditions revealed huge variation among different combinations of QTLs. The selection of NILs outperforming under different intensities of multiple stresses allows the selection of lines with desirable characteristics. The selected lines with significantly high grain yield advantage over Swarna under both NS as well as RS captured positive interaction due to both genotypic and phenotypic selection practised in the present study, a strategy suggested to be followed in marker assisted breeding for abiotic stress tolerance till all such unknown interactions are identified. Revealing of such epistatic interactions, between introgressed QTL and genetic background sheds light in understanding the differential phenotypic expression of introgressed/pyramided lines for quantitative traits that can be useful in future marker-assisted selection programs.

In our study, across ecosystems in India and Nepal, Swarna and Swana-Sub1 showed similar performance under drought at majority of the locations as against Swarna-Sub1 reported to show higher performance over Swarna under drought (Fukao et al. 2011).

The release of marker-assisted breeding product for drought earlier in IR64 background (Sandhu and Kumar 2017) and now in the present study in Swarna-Sub1 background are successful examples that should encourage breeders to use QTLs in the breeding programs targeting grain yield improvement under abiotic stresses. This is one of the first studies in rice developing stable and high-yielding varieties combining both drought and submergence tolerance in a high yielding variety background through marker-assisted backcrossing breeding successfully released as varieties for cultivation by farmers. In the present study, we observed improved performance of selected NILs over Swarna and Swarna-Sub1 under drought and submergence but we are yet to evaluate the lines under varying incidences of subsequent submergence and drought in the same season and observe plants adaptation. Even after affected by submergence under which duration of varieties increases by 10–12 days, the developed NILs showed an average of 10 days earlier flowering than Swarna-Sub1. This will allow the timely planting of second season crop hence enhancing sustainable productivity. The yield improvement across three different backgrounds, TDK-Sub1 (Dixit et al. 2017), IR64-Sub1 (data not published) and Swarna-Sub1 (present study) possessing both QTLs combining drought (DTYs) and submergence (*Sub1*) clearly indicates that the drought and submergence tolerance can be efficiently combined even though both have different molecular and physiological regulatory mechanisms. QTLs on grain yield under drought has been reported to effect water-uptake, stomatal conductance, canopy temperature, transpiration and root growth at depth (Henry et al. 2014, 2015) whereas the physiological evidence for the submergence tolerance points towards the proper balance between the production and consumption of plant assimilates (Singh et al. 2014; Kretzschmar et al. 2015), fast coleoptile elongation, expansin genes expression, and lower cell division and peroxidase activity (Magneschi and Perrata 2009). “The development and release of three drought-submergence tolerant varieties (CR dhan 801, Bahuguni dhan-1 and Bahuguni dhan-2) for cultivation in rainfed lowland areas of India and Nepal are successful examples of contribution of use of genomic tools to improve yield under drought and submergence”.

## Conclusions

Over last one-decade marker assisted breeding for abiotic stress has moved from just improving yield under drought or submergence to combining high yield potential with good yield under multiple stresses. The nine selected promising NILs with different QTL combination showed an average grain yield advantage of 0.2 to 1.7 t ha^− 1^ under RS and 0.1 to 1.0 t ha^− 1^ under submergence conditions with no yield penalty under NS. The three drought-submergence tolerant varieties CR dhan 801, Bahuguni dhan-1 and Bahuguni dhan-2 have been released for cultivation in India and Nepal. The marker-assisted derived drought and submergence tolerant rice varieties will help to reduce the yield losses associated with farming in drought-flood prone rainfed lowland areas, provide farmers with insurance of good yield and shall encourage marker-breeding programs developing better varieties tolerant to multiple abiotic and biotic stresses.

## Material and methods

### Plant material

Swarna, the popular rice variety, (released in 1979) and cultivated in 30–40% of rainfed lowland areas (~ 4.3 million ha) was chosen as a recipient to develop NILs using marker assisted backcrossing approach. Swarna-Sub1, the submergence tolerant NIL of Swarna was selected as the donor for *Sub1* gene. This variety is a long duration (140–145 days), medium-tall (95–100 cm), medium bold grain type with high tillering and an average yield of 6000–6200 kg ha^− 1^ was selected as donors for *Sub1*. Swarna-Sub1 provided yield advantage over Swarna under submergence condition. It has high head hulling percentage, high rice recovery and intermediate amylose content. Instead of traditional donors, advanced breeding lines possessing QTLs with high-yield potential were used in marker assisted breeding. IR 91659-54-35, an improved BC_3_F_3_ breeding line from the mapping population N22/Swarna possessing *qDTY*_*1.1*_, IR 81896-B-B-195, an improved BC_1_F_5_ line from Apo/Swarna population possessing *qDTY*_*2.1*_ and *qDTY*_*3.1*_ and Swarna-Sub1 possessing *Sub1* gene were used in the marker-assisted backcrossing program to combine three drought QTLs and *Sub1* gene in Swarna background.

### Development of NILs in Swarna-Sub1 background

**Fig. 5:**
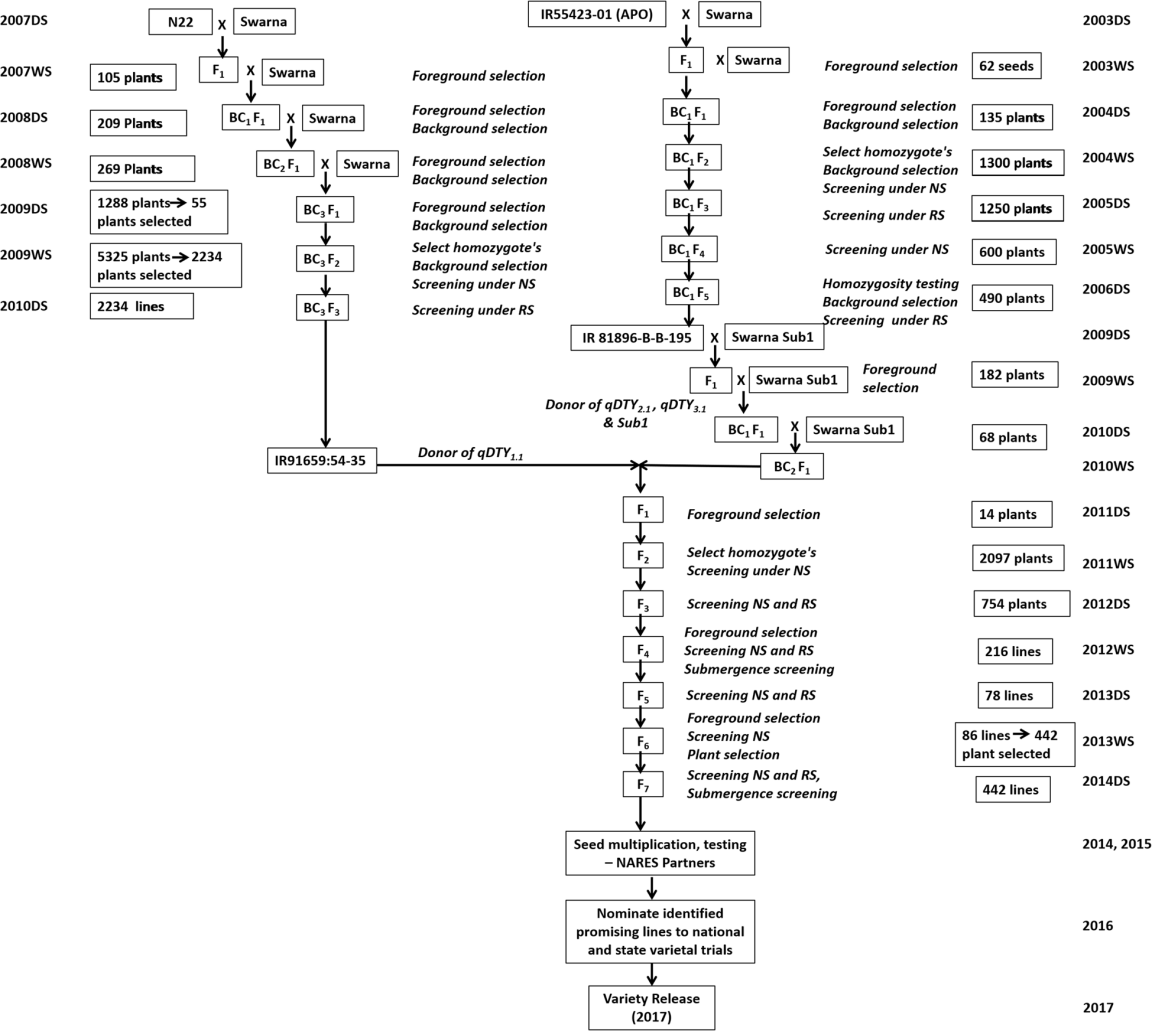
Detailed scheme for the development of Swarna-Sub1 NILs. Details on the foreground, background selection and the number of plants selected in every generation using marker assisted backcrossing breeding approach. DS: dry season, WS: wet season, NS: non-stress, RS: reproductive stage drought stress, NARES: National Agricultural Research and Extension Systems

The crossing scheme to develop NILs in Swarna-Sub1 background was initiated in 2009DS (DS: dry season), the NIL IR 81896-B-B-195, an improved BC_1_F_5_ line from Apo/Swarna population possessing QTLs *qDTY*_*2.1*_ and *qDTY*_*3.1*_ was crossed with (Swarna-Sub1), F_1_ was two times backcrossed with IR05F102 (Swarna-Sub1). In 2010WS (WS: Wet season), a BC_2_F_1_ plant possessing *qDTY*_*2.1*_, *qDTY*_*3.1*_ and *Sub*1 was crossed with IR 91659–54-35, an improved BC_3_F_3_ breeding line from the mapping population N22/Swarna possessing *qDTY*_*1.1*_. The backcrossed line IR 81896-B-B-195 used for introgression and pyramiding of *qDTY*_*2.1*_*, qDTY*_*3.1*_ QTLs came from the mapping population developed for identification of QTLs, from a crossing plan initiated in 2003DS (Fig. 5). The backcrossed line IR 91659-54-3-5 carrying *qDTY*_*1.1*_ was developed using N22 as donor, a crossing program initiated in 2007DS (Fig. 5). The detailed description on backcrossing, phenotypic plant selection, foreground, recombinant and background selection genotyping to identify NILs with desirable plant and grain type having introgressed QTLs is presented in Fig. 5. The markers linked to the QTLs targeted for introgression; *qDTY*_*1.1*_ (RM315, RM11943, RM431, RM12023, RM12091and RM12233), *qDTY*_*2.1*_ (RM5791, RM327, RM521, RM3549, RM324, and RM6374, RM424) and *qDTY*_*3.1*_ (RM15791, RM416, RM16030, RM520) and *Sub1* (ART5, SC3) were used for foreground selection in Swarna background.

### Description on experiments and agronomic management

The breeding lines were developed and screened under lowland transplanted control (non-stress; NS); lowland reproductive-stage drought stress conditions (RS) and seedling stage submergence condition (Sub). For evaluation of the introgressed lines and to identify the superior lines, a total of 60 experiments were conducted in Philippines (IRRI), Nepal (Hardinath, Nepalgunj) and India (Hyderabad, Sabour, Faizabad, Madhepura, Dhangain, Patna, Varanasi, Tripura, Cuttack, Hazaribagh and Raipur) from 2014DS to 2016WS. In all experiments each plot was 1 to 4 or more (advanced national trials) rows of 3 to 5 m plot length, with 0.20 m row-to-row spacing and 0.15-m plant to plant spacing. Nursery bed was raised and 21 to 25-days old seedlings were transplanted. The agricultural management practices were followed as Vikram et al. (2011).

### Screening for reproductive stage drought stress

The non-stress experiments were conducted under irrigated, flooded, puddled, transplanted, and anaerobic conditions with no drought and submergence stress. The reproductive-stage drought stress experiments referred to the experiments maintained as described by Sandhu et al. (2014). Depending on the site’s ability to measure data, tensiometers (at 30 cm depth), water pipes (1.1 m length) were installed and rainfall data was recorded across different years (Additional file 1: Figure S1). These measurements were collected from 50 to 100 days after seeding (DAS), which approximately represents the reproductive stage of the NILs evaluated under reproductive stage drought stress. For the reproductive stage drought stress, the stress was initiated at 50–52 days after seeding, after which the drought stress treatment was maintained depending on the rainfall. When the tensiometers reading ranged from − 50 to − 70 kPa, and the water table in PVC dropped to 100 cm from the soil surface and wilting and drying of leaves were observed (data not shown), the plots were re-irrigated. This cyclic screening at reproductive stage allows the precise screening of breeding lines with wide range of growth duration (Lafitte et al. 2004).

### Screening for submergence tolerance

The screening for submergence tolerance was carried out in Nepal (Hardinath, Nepalgunj) and India (Faizabad, Madhepura, Dhangain, Patna). The protocol for the screening for submergence tolerance was as described in Dixit et al. (2017). Selected NILs were planted in the nursery beds along with Swarna, Swarna-Sub1 and susceptible check IR42. The field was submerged for about two weeks from 14 days after seeding (DAS) to 27 days after seeding and then field was drained. The final recovery was recorded seven days after draining (Dixit et al. 2017). The tolerance to submergence was recorded on 1–9 scale of increasing order of susceptibility based on the standard evaluation system for submergence tolerance (IRRI 2002). In dry season, the submergence screening was conducted in a concrete tank facility. The selected lines were seeded in the seeding trays and at 14 DAS the trays were submerged along with IR42. The concrete tanks were drained on 30 DAS depending upon the survival of IR42, the susceptible check. The numbers of seedlings survived per line were recorded before submergence and at 2, 7, 14, and 21 days after draining the tanks, and percentage survival was calculated (Dixit et al. 2017). Survival percentage = (number of seedling survived after submergence/total number of seedlings planted)*100.

### Phenotypic evaluation

Key traits such as days to 50% flowering (DTF, days), plant height (PHT, cm), grain yield (GY, kg ha^− 1^) were measured and submergence was scored based on 1–9 scale. Days to 50% flowering referred to the day when more than 50% of the plants in plot showed panicle exertion. Plant height from root-shoot base to the highest panicle was recorded from three plants per plot and averaged. Grain yield data was recorded per plot and normalized for moisture content to 14% before final yield computation in kg ha^− 1^. Plant and panicle selections were made to get grain type similar to Swarna.

### Genotyping

Genotyping work was carried out at GSL (Genotyping Service Laboratory); IRRI Genomic DNA was extracted from leaves of 21 days old seedling using modified CTAB method (Murray and Thompson 1980).

The polymorphic markers linked to *qDTY*_*1.1*_ (RM315, RM11943, RM431, RM12023, RM12091, RM12146 and RM12233; Vikram et al. 2011), *qDTY*_*2.1*_ (RM5791, RM327, RM521, RM3549, RM324, and RM6374, RM424; Venuprasad et al. 2009) and *qDTY*_*3.1*_ (RM15791, RM416, RM16030, and RM520; Venuprasad et al. 2009) and *Sub1* (ART5; Septiningsih et al. 2009) was used for foreground selection in Swarna background. A total of 156 polymorphic markers out of a total of 600 were used for the background study. PCR (polymerase chain reaction) amplification was carried out to check and confirm the introgressed loci and the amplicon size was checked on non-denaturing 6% or 8% PAGE (polyacrylamide gel electrophoresis) depending on size of amplicon. SYBR SafeTM was used to stain the gel, viewed after 20 min, and allelic profile was recorded. Stepwise and precise selection involving both phenotyping and genotyping strategies were used to select and advance the plants with desired introgressed loci in every generation. The background genotyping of selected Swarna-Sub1 NILs was also done at GSL- IRRI using 6 K SNP Infinium chip.

### Evaluation of NILs in national trials

In India, in 2015, in All India Coordinate Rice Improvement Project (AICRIP), IR 96322-34-223-B-1-1-1-1 was evaluated along with 17 other introgressed lines and their respective recurrent parents, sensitive checks and donor parents of drought QTLs (IR 81896-B-B-195) in the Advance Variety Trial 1-Near Isogenic Line-Drought and Submergence’ (AVT1 NIL-Drt and Sub). The experiment was conducted at six locations (Cuttack, Pusa, Chinsurah, Titabar, Gerua and Ghagharaghat) to evaluate the entries under submergence and normal irrigated conditions. For drought stress as well as control conditions, trials were evaluated at four locations (ICAR-Patna, Varansai, Rewa and Coimbatore). In 2016, the entries that were promising during 2015 that includes IR 96322-34-223-B-1-1-1-1 along with recurrent parents, sensitive checks and donor parents of drought QTLs (IR 81896-B-B-195) were evaluated at eight locations (Maruteru, Chinsurah, Gerua, Moncompu, Pusa, NRRI, Ghaghraghat, Titabar) for submergence and at six locations (Gangavati, Masodha, Mugad, ICAR-Patna, Warangal and Coimbatore) for drought stress. At these locations, experiment was also conducted under normal irrigated conditions.

In both the years and at all locations in both stress and normal situations, the experiment was laid out in three replications in the randomized complete block design (RCBD), following the spacing of 20 × 15 cm in a plot size of 15 m^2^. Sowings under submergence were taken up in the last week of June and plantings in the last week of July to first week of August. Under normal irrigated conditions, sowings were taken up in the last week of June to first week of July and plantings in the last week of July to first week of August. In case of drought stress conditions, sowings were taken up from last week of June to second week of July and plantings from last week of July to second week of August. Fertilizers were applied as N:P_2_O_5_:K_2_O (90:30: 30) kg ha^− 1^ in control trials. In drought trials, fertilizer dose of 75:30:30 N:P_2_O_5_:K_2_O was applied. Observations were recorded for days to flowering, days to maturing, plant height (cm), biomass yield kg ha^− 1^, grain yield kg ha^− 1^, and harvest index. Under drought stress conditions, data was recorded on number of rain free days during crop growth season and stage of crop under drought stress was noted along with the severity of the stress. Protective irrigations were given at critical crop growth stages depending on the severity of the drought imposed as observed from the performance of susceptible checks and the recurrent parents. Under submergence stress, water stagnation of 30–80 cm was maintained.

A total of 46 Swarna-Sub1 + drought QTLs introgressed NILs were evaluated in three environments namely as control, reproductive drought stage stress and submergence at National Rice Research Program (NRRP, Hardinath, Nepal) during 2014 and 2015. The seeding date was June 19 in 2014 and June 18 in 2015. The twenty-one days old seedlings were transplanted in each trial. Two selected breeding lines, IR 96321–1447-651-B-1-1-2 and IR 94391–131–358-19-B-1-1-1 were evaluated in the National Coordinated Varietal Trials (NCVT) at Regional Agricultural Research Station, Tarahara, Parwanipur, Nepalgunj, and Lumle, National Maize Research Program, Rampur, National Wheat Research Program, Bhairahawa, under rainfed condition in 2015WS and 2016WS. All the experiments at NRRP Hardinath were laid out in alpha lattice with three replications where as in NCVT, the experiments were laid in RCBD design with two replications. The spacing was 20 cm between rows and 20 cm between plants. The plot size was 5 × 2 m^2^. Fertilizers were applied as N:P_2_O_5_:K_2_O (90:30: 30) kg ha^− 1^. In drought trials, fertilizer dose of 75:30:30 N:P_2_O_5_:K_2_O was applied. The submergence trial was subjected to 15 days complete submerge and then field was de-submerged. Seeding of drought stress trail was delayed by 25 days from normal planting so that rainy season terminated with the onset of reproductive stage of the crop. Trench was constructed around trial having the drought experiments to prevent seepage of water from other fields as well as for efficient drainage for drought imposition. In drought trials, tensiometer was installed to monitor soil moisture during the crop in the drought field. Pizzometer was also installed to monitor water table from the drought field. The observations on heading days, plant height (cm), and grain yield were recorded.

### Statistical analysis

#### Step 1: Single-trial analysis

Experimental designs across trials varied. For all the experiments with alpha lattice design, the effects of replications and blocks within replication were considered as random and lines as fixed. The model used for alpha lattice (AL) design was:

$$ {y}_{ijk}=\mu +{g}_i+{r}_j+{b}_{lj}+{e}_{ijk;} $$

For the Randomized Complete Block Design (RCBD) was:

$$ {\mathrm{y}}_{\mathrm{i}\mathrm{jk}}=\upmu +{\mathrm{g}}_{\mathrm{i}}+{\mathrm{r}}_{\mathrm{j}}+{\mathrm{e}}_{\mathrm{i}\mathrm{jk}} $$

For the augmented RCBD, the model used was:

$$ {\mathrm{y}}_{\mathrm{i}\mathrm{jk}}=\upmu +{\mathrm{g}}_{\mathrm{i}}+{\mathrm{b}}_{\mathrm{l}}+{\mathrm{e}}_{\mathrm{i}\mathrm{lk}} $$

where *μ* represents overall mean, *g*_*i*_ represents the effect of the *i*^*th*^ genotype, *r*_*j*_ represents the effect of the *j*^*th*^ replicate, *b*_*lj*_ represents the effect of the *l*^*th*^ block within the *j*^*th*^ replicate, *b*_*l*_ represents the effect of the *l*^*th*^ block and *e*_*ijk*_ is the error.

#### Step 2: Mean comparison of qDTY and Sub1 combinations

The performance of the breeding lines nested within the QTL class in the block within the replicate is modeled as follows:

$$ {\mathrm{y}}_{\mathrm{i}\mathrm{j}\mathrm{kl}}=\upmu +{\mathrm{r}}_{\mathrm{k}}+\mathrm{b}{\left(\mathrm{r}\right)}_{\mathrm{k}\mathrm{l}}+{\mathrm{q}}_{\mathrm{i}}+\mathrm{g}{\left(\mathrm{q}\right)}_{\mathrm{i}\mathrm{j}}+{\mathrm{e}}_{\mathrm{i}\mathrm{l}\mathrm{kl}} $$

where *μ* is the population mean, *r*_*k*_ is the effect of the *k*^*th*^ replicate, *b*(*r*)_*kl*_ + *q*_*i*_ is the effect of the *l*^*th*^ block within the *k*^*th*^ replicate, *q*_*i*_ is the effect of the *i*^*th*^ QTL, *g*(*q*)_*ij*_ is the effect of the *j*^*th*^ genotype nested within the *i*^*th*^ QTL and *e*_*ijkl*_ is the error. The effects of QTL and genotypes within QTL are considered fixed while the replicate and blocks within replicate effects are considered random. ANOVA and F test using SAS v9.2 were used to see whether the QTL classes differed significantly from each other.

#### Step 3: Q × E interaction model based on genotype means

Based on the trial mean grain yield, each experiment was re-classified for the observed drought stress intensity based on the yield reduction compared to non-stress (control) as per Kumar et al. (2009). The trials were classified as non-stress (control); moderate stress (30% to 65% yield reduction); severe stress (greater than 65%); and over-stressed (greater than 85%). Experiments which were classified as being overstressed were excluded from the analysis due to poor expression of their genetic variability.

The linear model used to study the Q *×* E interactions for the breeding lines tested across environment was:

$$ {\mathrm{y}}_{\mathrm{k}\mathrm{m}\mathrm{j}}=\upmu +{\mathrm{q}}_{\mathrm{k}}+\mathrm{g}{\left(\mathrm{q}\right)}_{\mathrm{k}\mathrm{j}}+{\mathrm{l}}_{\mathrm{m}}+{\mathrm{q}\mathrm{l}}_{\mathrm{k}\mathrm{m}}+\mathrm{g}{\left(\mathrm{q}\mathrm{l}\right)}_{\mathrm{k}\mathrm{m}\mathrm{j}}+{\mathrm{e}}_{\mathrm{k}\mathrm{m}\mathrm{j}} $$

Where *y*_*kmj*_ is the yield of the j^th^ breeding line nested in the k^th^ QTL class in the m^th^ environment, *μ* is the overall mean, *q*_*k*_ is the effect of the k^th^ QTL class, *g(q)*_*kj*_ is the effect of the j^th^ line nested in the k^th^ QTL class, *l*_*m*_ is the effect of the m^th^ environment, *ql*_*km*_ is the effect of the k^th^ QTL class in the m^th^ environment, *g(ql)*_*kmj*_ *g*(*ql*)_*kmj*_is the effect of the j^th^ line nested in the k^th^ QTL class in the m^th^ environment, ∈_*kmj*_ *e*_*kmj*_ is the random error of the j^th^ line nested in the k^th^ QTL class in the m^th^ environment. (Knapp 2001). All effects except the QTL were considered as random.

### Graphical representation of the selected NILs

Graphical representation of the background recovery of NILs using molecular marker data was performed in Graphical Genotypes (GGT 2.0) software (van Berloo 1999). The parent alleles of Swarna- *Sub1*, Apo, N22, and the heterozygous allele were scored as ‘S’, ‘A’, ‘B’, and ‘H’, respectively. The estimated proportion of the S, A, B, and H alleles in each NIL was calculated using the GGT 2.0.

## Additional file

Additional file 1 : **Table S1.** Detailed description on experiments with mean values of days to 50% flowering, plant height and grain yield across different experiments conducted at IRRI and at different location in India and Nepal between 2014 to 2016. **Table S2.** Comparison of QTL classes for mean grain yield (kg ha^− 1^) across generation advancement under irrigated NS and RS drought stress conditions at IRRI, Philippines. **Figure S1.** Rainfall data (mm) collected at different experimental sites during the reproductive stage drought stress screening in (A) 2014, (B) 2015, and (C) 2016. (DOCX 373 kb)
