# Supplementary material for: Marker Assisted Breeding to Develop Multiple Stress Tolerant Varieties for Flood and Drought Prone Areas
Source: Rice (N Y). 2019 Feb 18;12:8. doi: 10.1186/s12284-019-0269-y (PMC6379507; doi:10.1186/s12284-019-0269-y)
Supplement: Supplementary file 1 — : Table S1. Detailed description on experiments with mean values of days to 50% flowering, plant height and grain yield across different experiments conducted at IRRI and at different location in India and Nepal between 2014 to 2016. Table S2. Comparison of QTL classes for mean grain yield (kg ha− 1) across generation advancement under irrigated NS and RS drought stress conditions at IRRI, Philippines. Figure S1. Rainfall data (mm) collected at different experimental sites during the reproductive stage drought stress screening in (A) 2014, (B) 2015, and (C) 2016. (DOCX 373 kb) [file 12284_2019_269_MOESM1_ESM.docx]

**Additional file 1**

**Marker assisted breeding to develop multiple stress tolerant varieties for flood and drought prone areas**

Nitika Sandhu^1,2^, Shalabh Dixit^1^, BPM Swamy^1^, Anitha Raman^1^, Santosh Kumar^3^, S.P. Singh^4^, R.B. Yadaw^5^, O.N. Singh^6^, J.N. Reddy^6^, A. Anandan^6^, Shailesh Yadav^1^, Challa Venkataeshwarllu^7^, Amelia Henry^1^, Satish Verulkar^8^, N.P. Mandal^9^, T. Ram^10^, Jyothi Badri^10^, Prashant Vikram^1,11^, and Arvind Kumar^1*^

^1^ International Rice Research Institute, DAPO Box 7777, Metro Manila, Philippines

^2^ Punjab Agricultural University, Ludhiana, India

^3^ ICAR Research Complex for Eastern Region, Patna, Bihar, India

^4^ Bihar Agricultural University, Sabour, Bihar, India

^5^ National Rice Research Program Hardinath, Dhanusha, Nepal

^6^ ICAR-National Rice Research Institute, Cuttack, Odisha, India

^7^ International Rice Research Institute, South Asia Hub, ICRISAT, Patancheru, Hyderabad, India

^8^ Indira Gandhi Krishi Vishwavidyalaya, Raipur, Chhattisgarh, India

^9^ Central Rainfed Upland Rice Research station, National Rice Research Institute, Hazaribagh, Jharkhand, India

^10^ ICAR-Indian Institute of Rice Research, Hyderabad, India

^11^ International Maize and Wheat Improvement Centre (CIMMYT), Mexico

**^*^Corresponding author** : [a.kumar@irri.org](mailto:a.kumar@irri.org)

**Nitika Sandhu** : nitika.sandhu@gmail.com

**Shalabh Dixit** : [s.dixit@irri.org](mailto:s.dixit@irri.org)

**BPM Swamy** : [m.swamy@irri.org](mailto:m.swamy@irri.org)

**Anitha Raman** : [a.raman@irri.org](mailto:a.raman@irri.org)

**Santosh Kumar** : [santosh9239@gmail.com](mailto:santosh9239@gmail.com)

**SP Singh** : [sps2007bau2011@gmail.com](mailto:sps2007bau2011@gmail.com)

**R B Yadaw**  : rbaran_9@yahoo.com

**O.N. Singh** : [onsingh01@yahoo.com](mailto:onsingh01@yahoo.com)

**J.N. Reddy** : [jnreddycrri@gmail.com](mailto:jnreddycrri@gmail.com)

**A. Anandan** : anandanau@yahoo.com

**Shailesh Yadav** : [shailesh.yadav@irri.org](mailto:shailesh.yadav@irri.org)

**Challa Venkateshwarlu** : [c.venkateshwarlu@irri.org](mailto:c.venkateshwarlu@irri.org)

**Amelia Henry** : a.henry@irri.org

**Satish Verulkar** : [satishverulkar@gmail.com](mailto:satishverulkar@gmail.com)

**N. P. Mandal** : [npmandal@hotmail.com](mailto:npmandal@hotmail.com)

**T. Ram** : [t.ram2011@yahoo.com](mailto:t.ram2011@yahoo.com)

**Jyothi Badri** : jyothirishik@gmail.com

**Prashant Vikram** : pvikramseedwheat@gmail.com

**Arvind Kumar** : [a.kumar@irri.org](mailto:a.kumar@irri.org)

**Corresponding Author*

**Arvind Kumar**

Outcome Theme Leader- Resilient Rice

Plant Breeder, Rice Breeding Platform

International Rice Research Institute, DAPO Box 7777, Metro Manila, Philippines

**Table S1.** Detailed description on experiments with mean values of days to 50% flowering, plant height and grain yield across different experiments conducted at IRRI and at different location in India and Nepal between 2014 to 2016

| **Exp** | **Year/Season** | **Trt** | **Site** | **No. of Ent.** | **Experimental design** | **Rep** | **DTF**  **(days)** | **PHT**  **(cm)** | **GY (kg ha^-1^)** | **H** |
| --- | --- | --- | --- | --- | --- | --- | --- | --- | --- | --- |
| 1 | 2014DS | NS | IRRI-HQ | 426 | Augmented RCBD | - | 98 | 93 | 7878 | 0.78 |
| 2 | 2014DS | RS | IRRI-HQ | 426 | Augmented RCBD | - | 108 | 80 | 1652 | 0.62 |
| 3 | 2014WS | NS | IRRI-HQ | 48 | Alpha lattice (6x8) | 2 | 96 | 99 | 3309 | 0.77 |
| 4 | 2014WS | NS | IRRI-SAH | 45 | Alpha lattice (5x9) | 2 | 91 | 95 | 6186 | 0.52 |
| 5 | 2014WS | RS | IRRI-SAH | 45 | Alpha lattice (5x9) | 2 | 113 | 88 | 1003 | 0.54 |
| 6 | 2014WS | RS | Sabour | 44 | Alpha lattice (4x11) | 2 | 106 | 92 | 6739 | 0.58 |
| 7 | 2014WS | Sub | Faizabad | 44 | Alpha lattice (4x11) | 2 | 125 | 94 | 4670 | 0.95 |
| 8 | 2014WS | NS | Hardinath | 48 | Alpha lattice (4x12) | 2 | 108 | 86 | 2447 | 0.53 |
| 9 | 2014WS | RS | Hardinath | 48 | Alpha lattice (4x12) | 2 | 118 | 68 | 1072 | 0.52 |
| 10 | 2014WS | Sub | Hardinath | 48 | Alpha lattice (4x12) | 2 | 105 | 74 | 1290 | 0.36 |
| 11 | 2014WS | NS | IRRI-SAH | 18 | RCBD | 3 | 97 | 102 | 7150 | 0.81 |
| 12 | 2014WS | RS | IRRI-SAH | 18 | RCBD | 3 | 105 | 64 | 1208 | 0.96 |
| 13 | 2014WS | Sub | Faizabad | 18 | RCBD | 3 | 123 | 101 | 5122 | 0.96 |
| 14 | 2014WS | NS | IRRI-SAH | 7 | RCBD | 3 | 95 | 109 | 7203 | 0.44 |
| 15 | 2014WS | RS | IRRI-SAH | 7 | RCBD | 3 | 105 | 55 | 311 | 0.63 |
| 16 | 2014WS | NS | Sabour | 7 | RCBD | 3 | 98 | 92 | 8127 | 0.88 |
| 17 | 2014WS | NS | Madhepura | 7 | RCBD | 3 | 102 | 90 | 6984 | 0.73 |
| 18 | 2014WS | Sub | Madhepura | 7 | RCBD | 3 | 118 | 74 | 1711 | 0.66 |
| 19 | 2014WS | NS | Dhangain | 7 | RCBD | 3 | 93 | 92 | 7765 | 0.99 |
| 20 | 2014WS | Sub | Dhangain | 7 | RCBD | 3 | 92 | 92 | 7106 | 0.99 |
| 21 | 2014WS | Sub | Faizabad | 7 | RCBD | 3 | 128 | 99 | 4609 | 0.99 |
| 22 | 2014WS | NS | IRRI-SAH | 15 | Alpha lattice (3x5) | 3 | 91 | 106 | 7609 | 0.54 |
| 23 | 2014WS | RS | IRRI-SAH | 15 | Alpha lattice (3x5) | 3 | 96 | 54 | 1211 | 0.53 |
| 24 | 2014WS | NS | Sabour | 15 | Alpha lattice (3x5) | 3 | 86 | 100 | 5102 | 0.73 |
| 25 | 2014WS | NS | Madhepura | 15 | Alpha lattice (3x5) | 3 | 88 | 100 | 5102 | 0.77 |
| 26 | 2014WS | Sub | Madhepura | 15 | Alpha lattice (3x5) | 3 | 102 | 75 | 2903 | 0.86 |
| 27 | 2014WS | NS | Dhangain | 15 | Alpha lattice (3x5) | 3 | 83 | 95 | 7906 | 0.98 |
| 28 | 2014WS | Sub | Dhangain | 15 | Alpha lattice (3x5) | 3 | 83 | 96 | 8068 | 0.98 |
| 29 | 2014WS | Sub | Faizabad | 15 | Alpha lattice (3x5) | 3 | 98 | 98 | 5186 | 0.98 |
| 30 | 2015DS | NS | IRRI-HQ | 35 | Alpha lattice (5x7) | 2 | 116 | 86 | 6017 | 0.89 |
| 31 | 2015DS | NS | IRRI-SAH | 48 | Augmented RCBD | - | 95 | 87 | 6747 | 0.86 |
| 32 | 2015DS | RS | IRRI-SAH | 48 | RCBD | 2 | 98 | 69 | 1510 | 0.81 |
| 33 | 2015DS | Sub | Patna | 35 | RCBD | 2 | - | - | - | 0.87 |
| 34 | 2015WS | NS | IRRI-SAH | 23 | RCBD | 2 | 105 | 90 | 5968 | 0.37 |
| 35 | 2015WS | NS | Sabour | 16 | RCBD | 2 | 111 | 98 | 8375 | 0.21 |
| 36 | 2015WS | RS | Sabour | 16 | RCBD | 3 | 98 | 92 | 4140 | 0.64 |
| 37 | 2015WS | NS | Patna | 16 | RCBD | 3 | 106 | 99 | 7743 | 0.39 |
| 38 | 2015WS | RS | Patna | 16 | RCBD | 3 | 104 | 67 | 2987 | 0.92 |
| 39 | 2015WS | NS | Varanasi | 16 | RCBD | 3 | 114 | 88 | 9848 | 0.94 |
| 40 | 2015WS | NS | Tripura | 16 | RCBD | 3 | 97 | 99 | 4808 | 0.68 |
| 41 | 2015WS | NS | Hardinath | 24 | RCBD | 3 | 111 | 98 | 4523 | 0.54 |
| 42 | 2015WS | RS | Hardinath | 24 | RCBD | 3 | 102 | 71 | 2353 | 0.63 |
| 43 | 2015WS | Sub | Hardinath | 24 | RCBD | 3 | 85 | 118 | 1018 | 0.45 |
| 44 | 2015WS | NS | Nepalgunj | 24 | RCBD | 3 | 108 | 89 | 3756 | 0.44 |
| 45 | 2015WS | Sub | Nepalgunj | 24 | RCBD | 2 | 83 | 115 | 1278 | 0.4 |
| 46 | 2015WS | NS | IRRI-HQ | 48 | RCBD | 2 | 97 | 101 | 5222 | 0.80 |
| 47 | 2015WS | RS | IRRI-HQ | 48 | RCBD | 2 | 98 | 70 | 175 | 0.47 |
| 48 | 2015WS | NS | Hazaribagh | 16 | RCBD | 3 | 113 | 76 | 5652 | 0.89 |
| 49 | 2015WS | RS | Hazaribagh | 16 | RCBD | 3 | 107 | 67 | 3520 | 0.86 |
| 50 | 2016DS | NS | IRRI-HQ | 48 | Alpha lattice (4x4) | 2 | 85 | 84 | 2037 | 0.85 |
| 51 | 2016DS | RS | IRRI-HQ | 48 | RCBD | 2 | 89 | 59 | 605 | 0.50 |
| 52 | 2016WS | NS | Cuttack | 27 | Alpha lattice (3x9) | 2 | 93 | 104 | 4026 | 0.98 |
| 53 | 2016WS | NS | Patna | 27 | Alpha lattice (3x9) | 2 | 89 | 110 | 8921 | 0.9 |
| 54 | 2016WS | RS | Patna | 27 | Alpha lattice (3x9) | 2 | 85 | 99 | 5081 | 0.72 |
| 55 | 2016WS | RS | Raipur | 27 | RCBD | 2 | 86 | 93 | 1767 | 0.95 |
| 56 | 2016WS | NS | IRRI-HQ | 18 | Alpha lattice (3x6) | 3 | 87 | 108 | 4010 | 0.79 |
| 57 | 2016WS | NS | Hazaribagh | 16 | RCBD | 3 | 112 | 100 | 5940 | 0.81 |
| 58 | 2016WS | RS | Hazaribagh | 16 | RCBD | 3 | 116 | 85 | 4381 | 0.66 |
| 59 | 2016WS | NS | Nepalgunj | 52 | Alpha lattice (4x13) | 3 | 88 | 88 | 4756 | 0.30 |
| 60 | 2016WS | RS | Nepalgunj | 52 | Alpha lattice (4x13) | 3 | 93 | 75 | 1292 | 0.64 |

*Trt treatment, WS wet season, DS dry season, NS non-stress, RS reproductive stage drought stress, Sub submergence, Ent entries, Rep number of replications, IRRI HQ IRRI headquarter (Philippines), IRRI SAH IRRI South Asia Hub (Hyderabad), RCBD randomized complete block design, DTF days to 50% flowering (days), PHT plant height (cm), GY grain yield (kg ha^-1^), H heritability*

**Table S2** Comparison of QTL classes for mean grain yield (kg ha^-1^) across generation advancement under irrigated NS and RS drought stress conditions at IRRI, Philippines

| **QTL class** | **QTL** | **2012DS** | **2012DS** | **2012DS** | **2012DS** | **2012DS** | **2012WS** | **2013DS** | **2013DS** | **2014DS** | **2014DS** | **2015WS** | **2015WS** | **2016DS** |
| --- | --- | --- | --- | --- | --- | --- | --- | --- | --- | --- | --- | --- | --- | --- |
|  | Stress level | NS_Med | RS_Med | RS_ Med | NS_Late | RS_Late | NS | NS | RS | NS | RS | NS | RS | RS |
|  | Generation | F_3_ | F_3_ | F_3_ | F_3_ | F_3_ | F_4_ | F_5_ | F_5_ | F_7_ | F_7_ | F_8_ | F_8_ | F_8_ |
|  | Population Size | 663 | 366 | 304 | 91 | 84 | 754 | 432 | 432 | 432 | 432 | 48 | 48 | 48 |
| A | *qDTY_1.1_* | 4906 bc | 2677 cde | 2894 bcf | 6766 gh | 3674 c | 3925 bc | - | - | - | - | - | - | - |
| B | *qDTY_1.1_ + Sub1* | 5431 efg | 2228ab | 2930 bg | 4141 a | 3652 bc | 3536 bcd | - | - | - | - | 5191 c | 68.24 a | 579 c |
| C | *DTY_2.1_* | 4811cde | 2828 efg | 2962 abg | 4265 ab | 3719 bc | 4176 abc | - | - | - | - | - | - | - |
| D | *qDTY_2.1_ + Sub1* | 5084 cf | 2452 bcde | 2776 abde | 4649 ab | 3554 bc | 2729 a | 4109 bc | 793 bd | - | - | - | - | - |
| E | *qDTY_3.1_* | 5098 cdeg | 3010 gh | 3001 bg | 4987 ac | 2658 b | - | 4135 bc | 973 bd | 7941 ab | 1868 cd | - | - | - |
| F | *qDTY_3.1_ + Sub1* | 4705 bc | 3027 fh | 2984 bg | - | 3315 bc | 4663 ac | 4107 cd | 1097 be | 7934 b | 1838 cd | 4940 b | 97.96 a | 677 d |
| G | *Sub1* | 5430 cf | 2642 bcefh | 2334 ab | 5338bcd | 3204 bc | 3515 a | 2948 abc | 530 bd | - | - | - | - | - |
| H | *qDTY_1.1_+ qDTY_2.1_* | 5394 df | 2653 ce | 3131 efg | 6445 fg | 3671 c | 4308 ab | - | - | - | - | - | - | - |
| I | *qDTY_1.1_ + qDTY_2.1_ + Sub1* | 5444 ef | 2428 ac | 3133 efg | 6642 fgh | 3636 c | 4460 ab | 3710 bc | 605 bc | - | - | - | - | - |
| J | *qDTY_1.1_ + qDTY_3.1_* | 4788 c | 2693 de | 2945 be | 6395 fg | 3481 bc | 4288 ab | - | - | - | - | - | - | - |
| K | *qDTY_1.1_ + qDTY_3.1_ + Sub1* | 4989 cd | 2832 efg | 3003 ceg | 6639 efh | 3377 bc | 5183 c | 3456 b | 677 be | - | - | 4676 a | 159.19 b | 566 c |
| L | *qDTY_2.1_ + qDTY_3.1_* | 5265 bdf | 2998 fh | 2955 bg | - | 3620 bc | 4623 ac | 4116 cd | 992 cde | 7932 ab | 1672 bc | - | - | - |
| M | *qDTY_2.1_ + qDTY_3.1_ + Sub1* | 5154 cf | 3172 h | 3162 efg | 7380 hi | 3714 bc | - | 4192 cd | 1048 cde | 8194 b | 1503 ab | 5754 g | 360.16 c | 830 e |
| N | *qDTY_1.1_ + qDTY_2.1_ + qDTY_3.1_* | 5055 cd | 2845 df | 3130 dg | 7373 hi | 3505 c | 4807 bc | 3912 bd | 1073 d | 8043 b | 1854 d | - | - | - |
| O | *qDTY_1.1_ + qDTY_2.1_ + qDTY_3.1_ + Sub1* | 5484 ef | 3010 gh | 3167 fg | 6780 gh | 3859 c | 4838 bc | 4141 c | 1092 d | 8297 b | 1918 d | 5434 e | 356.81 c | 931 e |
| X | Swarna-Sub1 | 4683 ab | 2169 ab | 2673 a | 3749 a | 3169 ab | 5356 c | 2583 a | 304 a | 6388 a | 635 a | 5136 c | 129 a | 296 a |
| Y | Swarna | 3818 a | 2203 ab | 2465 a | 5827 cde | 2828 ab | 5146 c | 2106 a | 764 bd | 5818 a | 799 a | 5358 f | 64.45 a | 398 b |
| Trial mean | | 5077 | 2691 | 2937 | 6044 | 3474 | 4760 | 3615 | 838 | 7878 | 1652 | 5222 | 175 | 605 |
|  | F- value | 3.68 | 7.39 | 2.45 | 19.77 | 1.21 | 6.04 | 13.22 | 1.79 | 6.88 | 3.75 | 5.38 | 6.16 | 3.93 |
|  | p-value | 0.0168 | <.0001 | 0.0018 | 0.0001 | 0.2838 | <.0001 | 0.0003 | 0.0559 | 0.0003 | 0.0008 | <.0001 | 0.2991 | 0.368 |

The letters displayed are the QTL class labels ordered by increasing order of mean grain yield of QTL class, Means followed by same letter (within a column) are not significantly different, *DS dry season, WS wet season, NS non-stress, RS reproductive-stage drought stress, Med medium duration, Late late duration, X recipient parent (no QTL)*

*Source: Kumar et al. (2018)*


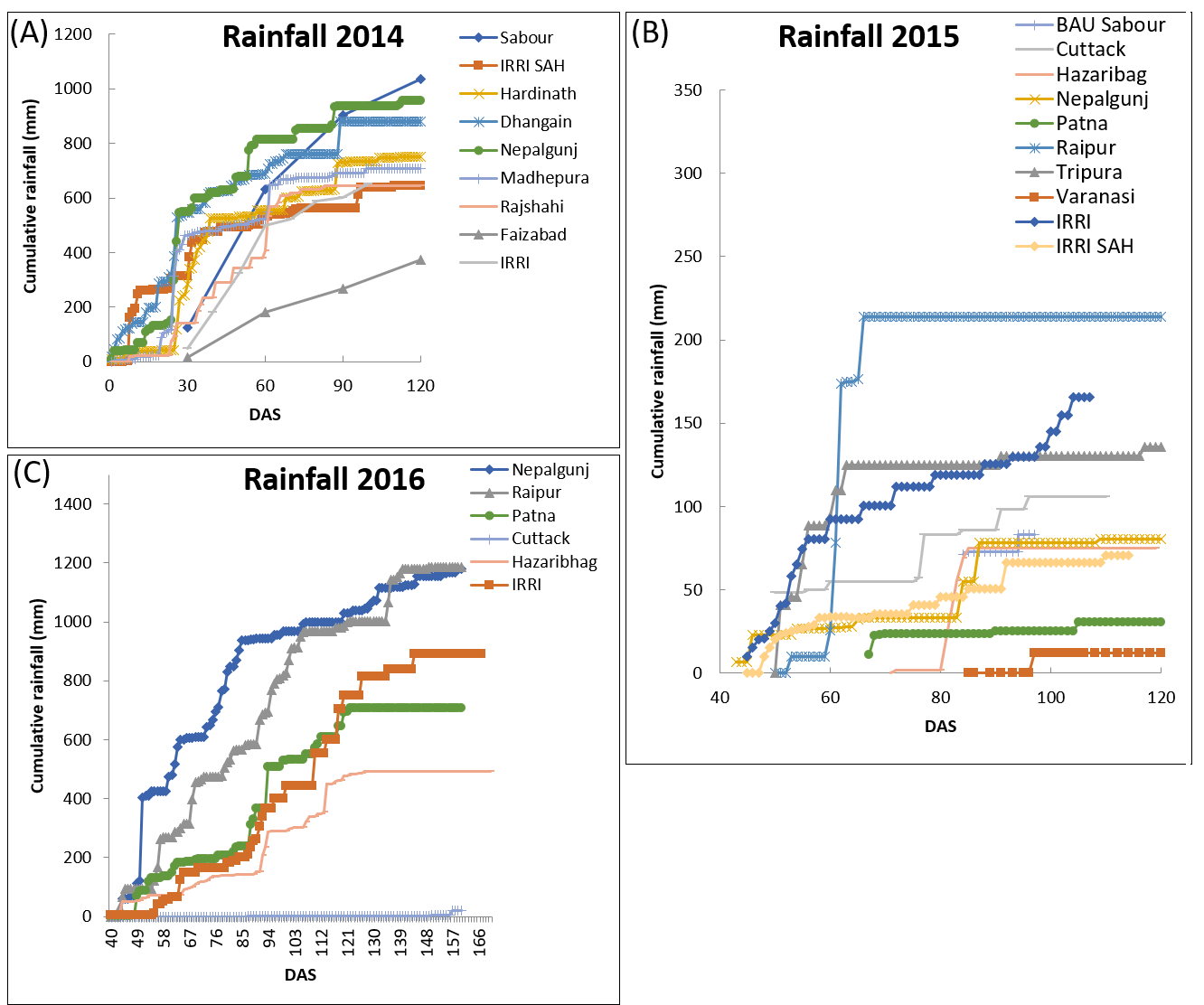


**Figure S1.** Rainfall data (mm) collected at different experimental sites during the reproductive stage drought stress screening in (A) 2014, (B) 2015, and (C) 2016

DAS: days after seeding, IRRI: International Rice Research Institute (Philippines), SAH: South-Asia Breeding Hub (Hyderabad)
